# The Etiology of Tuberculosis

**Published:** 1886-12

**Authors:** 


The Etiology of Tuberculosis.—Dugnet and Hèrcourt, of Paris, communicated to the Academy of Sciences, an account of three patients who died of tuberculosis running a rapid course. The affected organs were carefully examined and although tubercles were abundant, neither bacilli nor zooglea were detected, but in the neighborhood of the nodules there were found fine granulations, large spores and ramifications of mycelium like those seen in parasitic mycosis, *the microsporon furfur*, from which two of the patients had just recovered. Their investigations were then extended to other tubercular cases and they claim that these mycotic organisms are much more constant in tuberculosis than the bacillus. In fact they were never absent from tubercular granulations and were found in the caseous masses, which from some unknown causes are often free from the bacillus. The mycelia are abundant in the expectoration of consumptives where the bacillus tuberculosis is equally numerous. The mycelia also found in the excretions of patients clinically considered phthisical, in whom the bacillus is absent. The development of the bacillus tuberculosis from a parasite so abundant as the *microsporon furfur*, a parasite which often exists upon the person without apparent harm, is a matter of the greatest importance, which would be of especial value in prophylaxis. The author claims that the injection of the *microsporon furfur* in rabbits will produce tuberculosis. An Italian physician, Caragnis, however, injected two grams of water containing the epithelial scales taken from a patient with *pityriasis versicolor* into a rabbit. Fifty days after the animal was killed and found perfectly healthy.

Dr. A. Liebault, reports to the French Academy for the Advancement of Sciences, the results of his method of treating incontinence of urine by "*Hypnotic Suggestion.*" In this manner seventy-seven patients have been treated, varying in age from three years to adult life. Of these fifty-eight per cent. were males and forty-two per cent. females. The results obtained were exceedingly satisfactory.

1. Twenty-three patients have been cured by one or more seances and the malady has not returned.

2. Twenty-three were cured by the method but no reports have been received from them since the treatment has been discontinued.

3. Ten patients were cured by prolonged treatment and are still free from this trouble.

4. Marked improvement was observed in nine cases.

5. Four patients were treated but once and have not since been heard from.

6. Eight were neither improved nor cured. This report shows forty-three per cent. of certain cases. Among those treated were three persons of an advanced age who have entirely recovered.

---

According to the reports of the Registrar-General of Great Britain, the mortality rate of physicians is higher than that of those engaged in any other profession, in fact higher than the average of all occupations combined. The following are the figures giving the death-rate per 1,000:

| | |
|---|---|
| Clergymen, | 15.93 |
| School Teachers, | 19.90 |
| Lawyers, | 20.22 |
| Physicians, | 25.53 |
| All occupations, | 22.83 |

---

A Spanish physician, G. Andradas, recommends a two per 1,000 injection of bichloride of mercury for the treatment of gonorrhœal cystitis. An ounce and a half of this solution is injected and retained for three minutes. After two or three days an injection of larger quantity may be employed. The injection causes considerable pain on micturition.

---

During the year 1885–86 there were 14,633 students in the Italian universities. Of these, 5,195, or 35½ per cent., were medical students.

---

There are but three dispensaries in Paris. New York, with halt the population, has five times that number.

